# New Insights into the Benefits of Polyphenols in Chronic Diseases

**DOI:** 10.1155/2017/1432071

**Published:** 2017-11-19

**Authors:** A. Tresserra-Rimbau, S. Arranz, A. Vallverdu-Queralt

**Affiliations:** ^1^Department of Nutrition, Food Science and Gastronomy, XaRTA, INSA-UB, School of Pharmacy and Food Science, University of Barcelona, Barcelona, Spain; ^2^CIBEROBN Fisiopatología de la Obesidad y Nutrición, Instituto de Salud Carlos III, Madrid, Spain; ^3^AZTI-Tecnalia, Food Research Division, Derio, Bizkaia, Spain; ^4^INRA, UMR1083 Sciences pour l'œnologie, 2, Place Viala, 34060 Montpellier Cedex, France

A wide range of beneficial effects has been attributed to polyphenols. Several studies have shown an inverse association between polyphenols and chronic diseases such as stroke, cardiovascular diseases, metabolic syndrome, type 2 diabetes, and cancer. The Mediterranean diet and its key components, fruits and vegetables, wine, extra virgin olive oil, nuts, and also green tea and chocolate, are well known to contain a large amount of polyphenols. However, the protective effects of polyphenols *in vivo* depend on the extractability from food, intestinal absorption, metabolism, and biological action with target tissues. After the intake of different polyphenols, the metabolites present in plasma and urine vary from one person to another. This may be related to the different gut microbiota composition and/or genetic polymorphisms, and it may be linked to unambiguous health effects.

In this special issue, A. Tresserra-Rimbau et al. summarized the relation between polyphenols and stroke in human intervention studies, animal models, and *in vitro* studies, concluding that dietary polyphenols are inversely associated with morbidity and mortality due to cardio- and cerebrovascular diseases. This is important since the aforementioned diseases are a major concern worldwide.

A. M. Witkowska et al. assessed the link between the intake of dietary polyphenol and the incidence of cardiovascular disease in postmenopausal women. They reported that there was an inverse association between higher dietary polyphenol intake and cardiovascular diseases in this population. In the same line, B. A. Sandoval-Ramírez et al. reported that there is a direct interaction between the phenolic compounds present in beer, such as 8-prenylnaringenin, 6-prenylnaringenin, and isoxanthohumol, and intracellular estrogen receptors, which lead to gene expression modulation, increase in plasma concentrations of sex hormone, and thus modulation of physiological hormone imbalance in menopausal women. Thus, it urges to substitute traditional hormone therapies since they increase health risks. This, again, affirms the potential health benefits of polyphenol-rich food in women.

Metabolic syndrome is a cluster of medical conditions that raises the risk of having cardiovascular diseases and other health problems. According to G. Chiva-Blanch et al., it seems that polyphenols could improve metabolic syndrome components by decreasing body weight, blood pressure, and blood glucose and by improving lipid metabolism. However, evidence from long-term randomized trials is needed to develop public health strategies regarding metabolic syndrome. Based on the results of different studies regarding polyphenols and changes in menopause and metabolic syndrome, S. Kawvised et al. hypothesized that microencapsulated mulberry fruit extract, novel neuroprotectant, and memory-enhancing agent could protect brain damage and improve memory impairment in an animal model of menopause with metabolic syndrome.

Moreover, M. Guasch-Ferré et al. reported that polyphenols and also the Mediterranean diet and its key components are inversely associated to lower glycemia and type 2 diabetes incidence. Different mechanisms have been proposed such as promotion of glucose uptake in tissues and improvement of insulin sensitivity. Higher intake of flavan-3-ols and their food sources has shown beneficial effects, not only on insulin resistance but also on other cardiometabolic risk factors. Several prospective studies have also reached the same conclusions.

A. Riba et al. reported that resveratrol, a well-known stilbene mainly found in grapes and red wine, was able to prevent cardiac hypertrophy, contractile dysfunction, and remodeling when tested in some animal models of heart failure. A. Riba et al. were able to propose several mechanisms involved in its protective effects (i.e., inhibition of prohypertrophic signaling molecules, improvement of myocardial Ca^2+^ handling, regulation of autophagy, and reduction of oxidative stress and inflammation).

Lastly, A. Agouni et al. studied the vascular effects of dietary supplementation of a nonalcoholic red wine polyphenol extract in Zucker fatty obese rats. They found that the cyclooxygenase (COX) pathway was regulated by the wine polyphenol extract to maintain vascular tone within a physiological range.

In conclusion, this special issue highlights the importance of polyphenols in chronic diseases. Polyphenols from diets or specific foods provide health benefits through different mechanisms involving lipid and glucose metabolism, modifying gene expression or even influencing signaling pathways. However, their biological functions in humans still remain insufficient to claim clear and positive health effects relating to their consumption, particularly with regard to long-term dietary ingestion.

